# Electrophysiological Changes of Human-Induced Pluripotent Stem Cell-Derived Cardiomyocytes during Acute Hypoxia and Reoxygenation

**DOI:** 10.1155/2022/9438281

**Published:** 2022-12-19

**Authors:** Martta Häkli, Joose Kreutzer, Antti-Juhana Mäki, Hannu Välimäki, Reeja Maria Cherian, Pasi Kallio, Katriina Aalto-Setälä, Mari Pekkanen-Mattila

**Affiliations:** ^1^Heart Group, Faculty of Medicine and Health Technology, Tampere University, Tampere 33520, Finland; ^2^Micro- and Nanosystems Research Group, Faculty of Medicine and Health Technology, Tampere University, Tampere 33720, Finland; ^3^BioGenium Microsystems Oy, Tampere 33720, Finland; ^4^Heart Hospital, Tampere University Hospital, Tampere 33520, Finland

## Abstract

Ischemic heart disease is the most common cardiovascular disease and a major burden for healthcare worldwide. However, its pathophysiology is still not fully understood, and human-based models for disease mechanisms and treatments are needed. Here, we used human-induced pluripotent stem cell-derived cardiomyocytes (hiPSC-CMs) to model acute ischemia-reperfusion in our novel cell culture assembly. The assembly enables exchange of oxygen partial pressure for the cells within minutes, mimicking acute ischemic event. In this study, hypoxia was induced using 0% O_2_ gas for three hours and reoxygenation with 19% O_2_ gas for 24 hours in serum- and glucose-free medium. According to electrophysiological recordings, hypoxia decreased the hiPSC-CM-beating frequency and field potential (FP) amplitude. Furthermore, FP depolarization time and propagation slowed down. Most of the electrophysiological changes reverted during reoxygenation. However, immunocytochemical staining of the hypoxic and reoxygenation samples showed that morphological changes and changes in the sarcomere structure did not revert during reoxygenation but further deteriorated. qPCR results showed no significant differences in apoptosis or stress-related genes or in the expression of glycolytic genes. In conclusion, the hiPSC-CMs reproduced many characteristic changes of adult CMs during ischemia and reperfusion, indicating their usefulness as a human-based model of acute cardiac ischemia-reperfusion.

## 1. Introduction

Ischemic heart disease, the most common of cardiovascular diseases, is a major health burden worldwide and occurs when blood supply to cardiac tissue is inadequate [1]. Cardiac ischemia can occur suddenly (acute myocardial ischemia) when an artery supplying cardiac tissue becomes blocked due to a rupture of a plaque, which causes a blood clot and completely prevents blood supply to the cardiac tissue. Alternatively, ischemia can develop slowly when the artery builds up plaque over time narrowing the diameter of the artery lumen and reducing blood flow to the cardiac tissue (chronic myocardial ischemia) [2].

Currently, animal and primary animal cell models are the most frequently used models of ischemic heart disease for disease mechanisms, applications of therapeutic treatments, and drug development. However, one significant drawback of these models is the differences in human and animal physiology, which hinders the development of therapeutics for ischemic heart disease. Many drugs and treatments that have been promising in animal trials have failed in humans due to the different pathophysiological processes ongoing during the disease [3]. Therefore, efficient and reliable human-based models are required.

Human primary cardiomyocytes are difficult to harvest due to invasive biopsies and nonproliferative nature of adult cardiomyocytes [4], whereas human-induced pluripotent stem cells (hiPSCs) can be endlessly produced and differentiated into cardiomyocytes (CMs) [5]. However, hiPSC-derived CMs (hiPSC-CMs) are immature and resemble more fetal than adult CMs, limiting their utilization in several applications. For example, fetal and hiPSC-CMs are known to be more resistant to hypoxia, as they rely more on glucose for energy production while adult CMs use mainly fatty acid oxidation [6]. Nevertheless, over the past few years, researchers have started to utilize hiPSC-CMs in development of human-based ischemia-reperfusion models [6–11].

We have previously presented a platform for modeling cardiac ischemia-reperfusion [12], which allowed measuring hiPSC-CM electrophysiology with microelectrode array (MEA) technology and controlling and monitoring oxygen partial pressure in the cell culture simultaneously. However, our previous studies were designed to model chronic cardiac ischemia, and the oxygen level in the cell culture decreased gradually over four hours. On the other hand, acute ischemia due to a sudden coronary artery occlusion occurs much faster. In this study, we present a novel 1-well assembly with a modified lid structure providing the means to induce hypoxia to the cells in a matter of minutes and allowing the modeling of acute ischemia-reperfusion.

In the present study, we induced acute hypoxia and reoxygenation to hiPSC-CMs and evaluated their response to the oxidative stress. Real-time monitoring of the oxygen partial pressure (pO_2_) directly from the cell culture together with monitoring of the hiPSC-CM functionality long-term with the incorporated MEA system allows high-throughput data collection and comparison of the hiPSC-CM electrophysiological changes to the pO_2_ experienced by the cells. We show that the hiPSC-CM electrophysiology changes significantly during the acute hypoxia and reoxygenation, and the changes are similar to those known to occur in adult CMs during ischemia and reperfusion. Thus, the model of acute cardiac ischemia and reperfusion presented in this study can be a valuable tool for modeling mechanisms of the ischemic heart disease, as well as screening for new drugs and treatments for the ischemia-reperfusion injury in a human-based model.

## 2. Materials and Methods

### 2.1. Cell Culture and Cardiomyocyte Differentiation

Three control hiPSC lines were used in this study: UTA.04602.WT [13], UTA.10211.EURCCS [14], and UTA.11311.EURCCS (characterization protocol [15–17] and data presented in Supplementary Materials, Supplementary Figure S1). Before differentiation, the hiPSCs were maintained and expanded on mouse embryonic fibroblast feeder cells (Merck Millipore, Burlington, MA, USA) in KSR medium (KnockOut DMEM) (Gibco, Thermo Fisher Scientific, Waltham, MA, USA) containing 10% KnockOut Serum Replacement (Gibco), 1% MEM NEAA (Gibco), 1% GlutaMAX (Gibco), 0.2% *β*-mercaptoethanol (Gibco), and 0.5% penicillin/streptomycin (Lonza, Basel, Switzerland). The embryoid body differentiation was used to acquire cardiomyocytes as described previously [18].

### 2.2. MACS and hiPSC-CM Seeding

Magnetic-activated cell sorting (MACS) was performed on day 20 of differentiation as described previously [19, 20]. Multi Tissue Dissociation Kit (Miltenyi Biotec, Bergisch Gladbach, Germany) was used for dissociating the embryoid bodies into single cells, and PSC-Derived Cardiomyocyte Isolation Kit (human) (Miltenyi Biotec) was used to isolate the CMs. The CMs were suspended in 20% EB medium (KnockOut DMEM containing 20% FBS (Gibco), 1% MEM NEAA, 1% GlutaMAX, and 0.5% penicillin/streptomycin) and seeded as cell sheets (~95 000 cells/cm^2^) on 1-wells on glass or microelectrode array (MEA) plates (60MEA200/30iR-Ti, 8 × 8) (Multi Channel Systems MCS GmbH, Reutlingen, Germany) coated with 2% Geltrex (Gibco) in KnockOut DMEM. Before hypoxia and reoxygenation, the CMs were cultured for eight days. The samples were kept in Petri dishes in a normal incubator without the lids and covers, and half of the culture medium was exchanged three times a week.

### 2.3. Acute Lid

**Figure 1:**
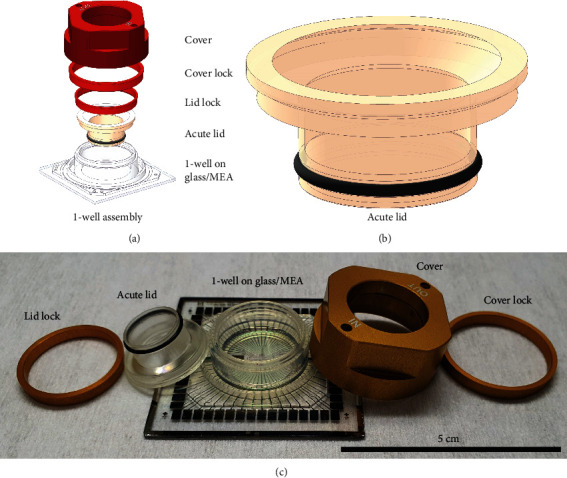
1-well assembly and acute lid. (a) Illustration of the 1-well assembly. The Acute lid is attached to 1-well assembly and sealed against the silicone injection moulded 1-well structure with a lid lock. The assembly is covered with a cover that is locked tightly to the assembly with a cover lock. (b) Illustration of the acute lid. The acute lid consists of a hollow cylinder covered with a gas permeable polyethylene membrane attached with an O-ring. (c) A real image of the 1-well assembly and the acute lid (scale bar 5 cm).

The platform for inducing hypoxia and reoxygenation for hiPSC-CMs was similar to the system previously used for modeling chronic ischemia in hiPSC-CMs [12, 20], except the lid was changed from a solid glass-made lid to a hollow lid covered with a gas-permeable membrane. This acute lid design (Figure 1) allows rapid changes of oxygen partial pressure in the samples due to the proximity (500 *μ*m) of the gas-permeable membrane to the cells. The acute lid was 3D printed from cytocompatible dental resin using Form 3 3D printer (Formlabs Inc., Somerville, MA, USA). The gas-permeable polyethylene membrane was attached tightly with an O-ring around the hollow cylinder in the lid.

The technical performance of the acute lid was characterized by measuring the dynamics of the oxygen partial pressure with and without cells and by determining the evaporation from the 1-wells. A step response from 19 kPa to 0 kPa pO_2_ was measured for three individual acute lids, twice for each lid. Fall time (time between 90% and 10% of the step) of the step response was calculated for all measurements. A custom-made oxygen sensor platform [21] was utilized for the measurements.

The evaporation rate through the thin membrane was determined to demonstrate the compatibility of the lid to be used with dry gas mixture supply (this is an important aspect as no humidification of gas supply is needed). Evaporation studies were performed only without the cells with dH_2_O in 1-well assemblies on glass in OxyGenie platform (The Baker Company, Sanford, ME, USA). Reference 1-wells on glass were kept in a standard incubator inside Petri dishes (two 1-wells on glass inside one Petri dish) without the acute lid and the cover. Evaporation was determined by measuring the weight of the assemblies in the beginning and at the end of the experiment.

### 2.4. Hypoxia and Reoxygenation

**Figure 2:**
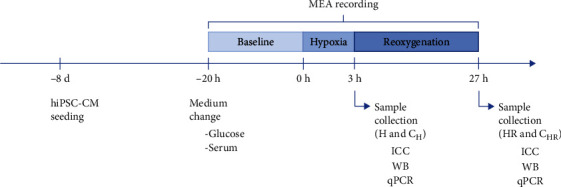
Timeline of the experiments. hiPSC-CMs were seeded on 1-wells on glass or MEA 8 days before hypoxia start. Glucose- and serum-free medium was exchanged to the samples on the day before the hypoxia start. 0% O_2_ hypoxia was induced for 3 hours, after which samples (H) were collected for immunocytochemistry (ICC), western blot (WB), and qPCR, or 19% O_2_ reoxygenation was induced to the samples. After 24-hour reoxygenation, samples (HR) were collected for ICC, WB, and qPCR. Control samples were kept in a standard incubator for the duration of the hypoxia (C_H_) or hypoxia-reoxygenation (C_HR_). For MEA samples, baseline was recorded in 19% O_2_ after medium exchange, after which 0% O_2_ hypoxia and 19% O_2_ reoxygenation were induced as for other samples.

The experimental timeline is presented in Figure 2. Hypoxia and reoxygenation experiments were performed after 30 days of differentiation. Serum- and glucose-free EB medium (glucose-free DMEM (Gibco) containing 1% MEM NEAA, 1% GlutaMAX, and 0.5% penicillin/streptomycin) was exchanged to both hypoxia and control samples day before inducing hypoxia to the samples. Glucose-free medium was used, as it has been shown that the presence of glucose can protect CMs from oxidative stress [22].

Hypoxia and reoxygenation of the 1-well assemblies on MEA were induced by connecting the samples on the MEA recording system to 19% O_2_, 5% CO_2_, and 76% N_2_ (normoxic) gas for overnight baseline measurement, 0% O_2_, 5% CO_2_, and 95% N_2_ (hypoxic) gas for three-hour hypoxia, and 19% O_2_, 5% CO_2_, and 76% N_2_ gas for 24-hour reoxygenation. Hypoxia (H) and reoxygenation (HR) of the 1-well assemblies on glass were induced using a portable OxyGenie platform, which allows simultaneous exposure of six parallel samples [12, 20, 23–25]. Hypoxia was induced for three hours using hypoxic gas, whereas reoxygenation was induced for 24 hours using normoxic gas. The control samples were kept in the incubator inside Petri dishes (two 1-wells in one Petri dish) without the acute lids and covers for the duration of the experiment (C_H_ for the duration of the hypoxia and C_HR_ for the duration of the hypoxia-reoxygenation). After the experiment, the samples were collected one by one to minimize the exposure to ambient air.

### 2.5. Measurement of Oxygen Partial Pressure

The oxygen partial pressure (pO_2_) was measured from six samples without cells and from five MEA samples with cells to evaluate the oxygen dynamics in the samples and to compare the pO_2_ to the electrophysiological changes of the hiPSC-CMs during hypoxia and reoxygenation. A luminescence-based sensor and a biocompatible sensing material were used for measuring the pO_2_ every 15 seconds for the duration of the experiment [21, 26, 27]. After the experiment, the cells were detached from the MEA, and the oxygen measurements were calibrated with the same MEA without the cells.

### 2.6. Field Potential Recording and Analysis

hiPSC-CM field potentials were recorded using microelectrode arrays (60MEA200/30iR-Ti, 8 × 8 (electrode diameter of 30 *μ*m and interelectrode spacing of 200 *μ*m) ordered from Multi Channel Systems MCS GmbH) to evaluate electrophysiological response of the hiPSC-CMs to hypoxia and reoxygenation. The basic electrophysiological characterization of the hiPSC-CMs used in this study has been previously performed [18]. MEA samples without pO_2_ measurement were placed on MEA2100 System (Multi Channel Systems MCS GmbH) and connected to normoxic gas for overnight baseline measurement, hypoxic gas for 3-hour hypoxia, and normoxic gas for 24-hour reoxygenation. MEA measurements were performed with Multi Channel Experimenter (version 2.17.5.21056, Multi Channel Systems MCS GmbH) using 25 kHz sampling frequency. Baseline was recorded overnight (at least 18 hours) for 1 min every 30 minutes but changed to record 1 min every 5 minutes one hour before initiating the hypoxia. The recording during hypoxia was done for 1 min every other minute for the first 30 minutes, 1 min every 5 minutes for the following 30 minutes, and 1 min every 10 minutes for the remaining two hours. For the first three hours of reoxygenation, the recording protocol was the same as with hypoxia, and after that, 1 min every 30 min was recorded for the remaining 21 hours.

MEA samples with pO_2_ measurement were placed on the MEA2100 Lite System (Multi Channel Systems MCS GmbH) and the measurements were performed with Multi Channel Experimenter (version 2.17.8.21078) using 20 kHz sampling frequency. Baseline was recorded overnight (at least 18 hours) for 1 min every 30 minutes but changed to record for 1 min every 5 minutes one hour before initiating hypoxia. The recording during the three-hour hypoxia as well as the first three hours of reoxygenation was done for 1 min every 5 minutes. For the remaining 21 hours of reoxygenation, 1 min every 30 min was recorded. Phase-contrast images were taken from all MEA samples before and after the experiment with Axio Observer (ZEISS, Oberkochen, Germany) using EC Plan-Neofluar 10 x/0.30 Ph1, WD 5.2 mm objective (ZEISS). The images were taken with Axiocam 506 color camera (ZEISS).

Beating frequency (BPM), field potential duration (FPD), field potential amplitude (*A*_dep_), and depolarization time (*t*_dep_) were analyzed from 22 MEA samples. Five electrodes were chosen from each sample for analysis based on strong baseline activity (altogether 110 electrodes). The raw data were converted from MSRD to HDF5 format with Multi Channel DataManager (version 1.12.0.20014, Multi Channel Systems MCS GmbH), and the data were then analyzed with MATLAB (version R2019b, MathWorks, Inc., Natick, MA, USA) using in-house developed scripts. Field potential duration was beating rate-corrected (corrected field potential duration, cFPD) using Izumi-Nakaseko formula [28].

Field potential propagation (FPP) through the hiPSC-CM sheets was analyzed from four samples. Peak timestamps were detected from the recorded files using Multi Channel Analyzer (version 2.14.0.19346, Multi Channel Systems MCS GmbH), and R (version 4.0.2, R Foundation for Statistical Computing, Vienna, Austria) was used to calculate the field potential propagation between adjacent electrodes based on the timestamps of each FP.

### 2.7. Immunocytochemical Staining

hiPSC-CM morphology and protein expression were evaluated from fluorescent images of immunocytochemical (ICC) staining of the 1-well assemblies on glass. The samples were fixed in 4% paraformaldehyde for 20 min, blocked in 10% normal donkey serum (Biowest, Nuaillé, France) solution for 45 min, and stained against cardiac troponin T (cTnT), cardiac myosin binding protein C (cMyBP-C), and hypoxia inducible factor 1 alpha (HIF1-*α*) in primary antibody solution containing goat anti-troponin T (ab64623, 1 : 1000, Abcam, Cambridge, UK), mouse anti-MyBPC3 (sc-166081, 1 : 400, Santa Cruz Biotechnology, Dallas, TX, USA), and rabbit anti-HIF1*α* (700505, 1 : 500, Invitrogen, Waltham, MA, USA) at 4°C overnight (ON). Donkey anti-goat Alexa Fluor 568, donkey anti-mouse Alexa Fluor 647, and donkey anti-rabbit Alexa Fluor 488 (1 : 800, Thermo Fisher Scientific) were used as secondary antibodies (1-hour incubation at room temperature (RT)). Vectashield Mounting Medium with DAPI (Vector Laboratories, Burlingen, CA, USA) was used to counterstain the cell nuclei. The fluorescence was visualized with an Olympus IX51 Microscope (Olympus, Tokyo, Japan) using LUCPlan FL N 40x/0.60, WD 3.0–4.2 mm (air) objective (Olympus). Images of the fluorescent stainings were taken with Orca Flash4.0LT+sCMOS camera (Hamamatsu Photonics, Hamamatsu, Japan). Confocal images were taken with LSM 800 Laser Scanning Confocal Microscope (ZEISS) using EC Plan-Neofluar 40x/0.75, WD 0.71 mm (air) objective (ZEISS), and 2 channel spectral detection with high-sensitivity PMT detector.

### 2.8. Nucleus Area Analysis

The area of the nuclei was determined from the DAPI channel of the fluorescent images of the ICC staining using ImageJ software [29]. The images were converted to binary by automatic thresholding, and watershed was used to estimate nuclei borders in aggregates. Analyzed particle command was used to determine the nucleus area so that nuclei crossing the image border were excluded, as well as nuclei with an area smaller than 30 *μ*m^2^ or larger than 400 *μ*m^2^.

### 2.9. Western Blot

hiPSC-CM HIF1-*α* expression was evaluated by western blot from the protein samples collected from 1-well assemblies on glass. Protein samples were collected in 150 *μ*l of 2× Laemmli Sample Buffer (Bio-Rad, Hercules, CA, USA) containing 5% *β*-mercaptoethanol (Sigma-Aldrich, Saint Louis, MO, USA) heated at 95°C for 5 min and stored at -20°C. 20 *μ*l of the protein samples was run in 4-20% Mini-PROTEAN TGX Precast Protein Gel with 10 50 *μ*l wells (Bio-Rad), and the samples were blotted to PVDF membranes using Trans-Blot Turbo RTA Mini PVDF Transfer Kit (Bio-Rad) with Trans-Blot Turbo Transfer System (Bio-Rad). The membranes were blocked with 3% BSA (Sigma-Aldrich) for three hours and incubated in primary antibody solution at 4°C ON. Mouse anti-*β*-actin (sc-47778, 1 : 1000, Santa Cruz Biotechnology) and rabbit anti-HIF1*α* (700505, 1 : 1000, Invitrogen) were used as primary antibodies. Horseradish peroxidase-conjugated anti-mouse IgG (sc-516102, 1 : 3000, Santa Cruz Biotechnology) and anti-rabbit IgG (P0217, 1 : 2000, DAKO, Agilent Technology, Santa Clara, CA, USA) were used as secondary antibodies (1-hour incubation at RT). The protein-antibody complexes were detected using Amersham ECL Prime Western Blot Detection Reagent (Cytiva, Marlborough, MA, USA), and ChemiDoc MP Imaging System (Bio-Rad) was used for imaging. The antibodies were stripped in stripping buffer (0.705% *β*-mercaptoethanol, 2% SDS (Sigma-Aldrich), and 0.03125 M Tris (VWR, Radnor, PA, USA) in Milli-Q water) at 56°C for 30 min before new blocking and antibody incubation.

### 2.10. Gene Expression

hiPSC-CM gene expression was analyzed using RT-qPCR. RNA samples were collected from 1-well assemblies on glass using Monarch Total RNA Miniprep Kit (New England BioLabs, Ipswich, MA, USA) and following the kit instructions. RNA was eluted two times in 20 *μ*l of nuclease-free (NF) water (final volume 40 *μ*l). High-capacity cDNA Reverse Transcription Kit (Applied Biosystems, Waltham, MA, USA) was used for reverse transcription following the kit instructions.

Due to low RNA amount, preamplification was performed using TaqMan PreAmp Master Mix (Applied Biosystems) following the kit instructions. The samples were run with the following thermal cycler protocol: 10 min at 95°C, 10 cycles of 15 sec at 95°C, and 4 min at 60°C, and at 4°C until the samples were stored at -20°C.  Table 1 shows the 20× TaqMan assays (Applied Biosystems) used in the preamplification and qPCR.

To analyze the *PKM* isoform expression in hiPSC-CMs, custom assays specific for *PKM* isoforms 1 and 2 were designed, as commercially available 20× TaqMan assays did not exist. Exon 7 and 8 regions with the most sequence variation were targeted for the design of primers and probe. All primer/probe sequences were imported into the custom TaqMan assay design software tool (Thermo Fisher Scientific) to generate the custom assays. The probes were designed to incorporate a minor groove-binding moiety (MGB) and were labeled with a fluorescent dye (FAM) for detection and a nonfluorescent quencher. Primers and probes were custom ordered from Thermo Fisher Scientific. Sequences for the primer/probe combinations are presented below.

(1) *PKM* isoform 1:
  - Forward primer: 5′-CAGCACCTGATAGCTCGTGA-3′
  - Reverse primer: 5′-TCAAAGCTGCTGCTAAACACTT-3′
  - Probe: 5′-AACTTGTGCGAGCCTCAAGT-3′
(2) *PKM* isoform 2.
  - Forward primer: 5′-AGAAACAGCCAAAGGGGACT-3′
  - Reverse primer: 5′-CACTGCAGCACTTGAAGGAG-3′
  - Probe: 5′-CTGCCATCTACCACTTGCAA-3′

TaqMan Fast Advanced Master Mix (Applied Biosystems) was used for qPCR following kit instructions, and three technical replicates for each gene and sample were used. The preamplified cDNA samples were diluted at 1 : 5 (20 *μ*l of sample in 80 *μ*l of NF water). One reaction contained 2 *μ*l of diluted preamplified cDNA, 0.4 *μ*l of 20× TaqMan assay, and 4 *μ*l of Fast Advanced Master Mix (total reaction volume 8 *μ*l). The qPCR plates were run with the following protocol: 2 min at 50°C, 2 min at 95°C and 40 cycles of 3 sec at 95°C, and 30 sec at 60°C. The 2^−ΔΔCt^ method was used to calculate the relative expressions of the genes using hypoxia control samples for normalization [30]. *GAPDH*, *EEF1A1*, and *TBP* were used as endogenous controls.

### 2.11. Statistical Analysis

Statistical analysis of the data was performed using rstatix (version 0.7.0) [31] in R. The Pairwise Wilcoxon signed-rank sum test with Bonferroni correction was used for BPM, *t*_dep_, *A*_dep_, and cFPD. The Kruskal-Wallis test was used for multiple comparisons, and Dunn's test with Bonferroni correction was used as a post hoc test for pairwise comparisons for FPP, relative gene expression, nucleus size, and western blot data. Sample numbers and differentiation batches for each cell line and experiment type are presented in Supplementary Table S1.

## 3. Results

### 3.1. Acute Lid and Oxygen Partial Pressure

**Figure 3:**
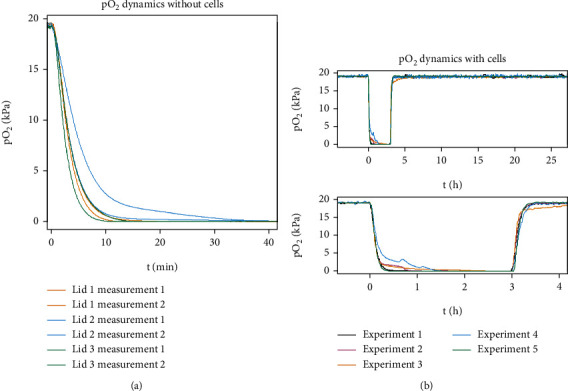
The dynamics of the oxygen partial pressure (pO_2_) in 1-well assembly with and without the hiPSC-CMs. (a) Oxygen partial pressure measurements from 19 kPa to 0 kPa pO_2_ without cells (*n* = 6). The mean fall time (from 90% to 10%) was 6.35 ± 2.55 min. (b) Oxygen partial pressure measurements from 19 kPa to 0 kPa pO_2_ with cells on MEA (*n* = 5). The mean fall time was 18.97 ± 15.86 min.

In this study, 1-well assemblies (Figures 1(a)–1(c)) were used with acute lids (Figures 1(b) and 1(c)) to achieve fast changes in the oxygen partial pressure in cell culture. pO_2_ measurements with the acute lid were performed using normoxic gas (baseline and reoxygenation) and hypoxic gas (hypoxia) demonstrating fast oxygen dynamics. The fall time (from 90% to 10%) was 6.28 ± 2.28 min without the cells (*n* = 6) (Figure 3(a)) and 18.97 ± 15.86 min measured from MEA samples with the cells (*n* = 5) (Figure 3(b)); however, the initial drop in pO_2_ is faster, and fall from 90% to 30% (5 kPa) pO_2_ is achieved already in 7.65 ± 2.34 min. The rise time (from 10% to 90%) measured from MEA samples with the cells during reoxygenation was 12.61 ± 1.94 min (*n* = 5) (Figure 3(b)).

The liquid evaporation was measured without cells from 1-well assemblies with acute lids (*n* = 12) and reference 1-wells on glass without acute lids in an incubator (*n* = 12). The measured evaporation was 1.5 *μ*l/h ± 1.1 *μ*l/h for 1-well assemblies with acute lids and 2.8 *μ*l/h ± 0.2 *μ*l/h for the reference wells, indicating that the use of nonhumidified gas with the 1-well assemblies does not cause high liquid evaporation.

### 3.2. Electrophysiological Changes of hiPSC-CM during Hypoxia and Reoxygenation

**Figure 4:**
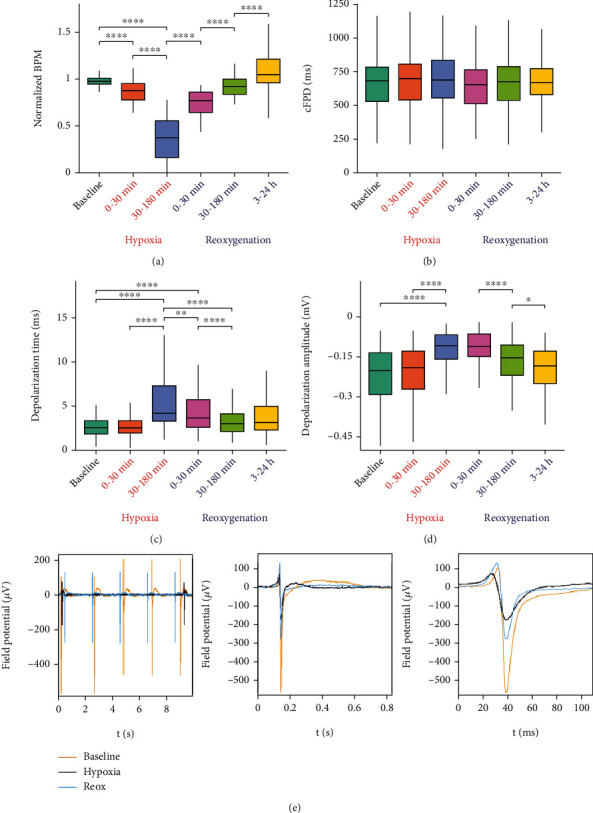
Functionality of the hiPSC-CMs was evaluated via MEA measurements during baseline, hypoxia, and reoxygenation. (a) Normalized beating frequency (beats per minute, BPM) of hiPSC-CMs decreased during hypoxia and was restored during reoxygenation. (b) Corrected field potential duration (cFPD) did not change significantly during hypoxia or reoxygenation. (c) Depolarization time of the hiPSC-CMs increased during hypoxia and returned close to baseline during reoxygenation. (d) hiPSC-CM depolarization amplitude decreased significantly during hypoxia and recovered during reoxygenation. (e) Examples of a filtered FP signals acquired from MEA recordings during baseline (yellow), hypoxia (black), and reoxygenation (blue). ^∗^*p* < 0.05, ^∗∗^*p* < 0.01, ^∗∗∗^*p* < 0.001, and ^∗∗∗∗^*p* < 0.0001.

**Figure 5:**
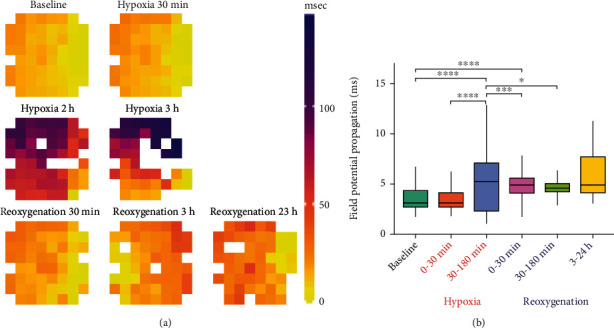
Field potential propagation (FPP) through the hiPSC-CM sheet during baseline, hypoxia, and reoxygenation. (a) Propagation of the FP the hiPSC-CM sheet on the MEA electrodes during baseline and different hypoxia and reoxygenation timepoints. (b) Hypoxia significantly increased the FPP time calculated between adjacent electrodes, whereas reoxygenation decreased it. However, even at the end of the reoxygenation period, the FPP remains increased compared to the baseline. ^∗^*p* < 0.05, ^∗∗∗^*p* < 0.001, and ^∗∗∗∗^*p* < 0.0001.

hiPSC-CM electrophysiology was evaluated using MEA technology for recording field potentials from the hiPSC-CM sheets during baseline, hypoxia, and reoxygenation. The recordings were evaluated for beating frequency (beats per minute, BPM) (Figure 4(a)), beating rate-corrected field potential duration (cFPD) [28] (Figure 4(b)), depolarization time (*t*_dep_) (Figure 4(c)), depolarization amplitude (*A*_dep_) (Figure 4(d)), and field potential propagation (FPP) throughout the hiPSC-CM sheet (Figure 5).

Beating frequency of the hiPSC-CMs decreased significantly during hypoxia (Figures 4(a)–4(e), Supplementary Figure S2). The beating frequency was normalized for each sample to the mean value of the three baseline values just before initiating hypoxia to get comparable results despite differences in the baseline-beating frequency between the samples. The absolute BPM values are presented in Supplementary Figure S2 for all samples together and by cell line. BPM of the hiPSC-CMs dropped already during the first 30 minutes of hypoxia and further decreased and often ceased altogether until reoxygenation was started. Reoxygenation restored the hiPSC-CM beating quickly, but the mean BPM increased even after the first 3 hours of reoxygenation. The mean normalized BPM was 0.94 ± 0.16 during baseline, 0.85 ± 0.17 during 0-30 min hypoxia (*p* < 0.0001 vs. baseline), 0.35 ± 0.23 during 30-180 min hypoxia (*p* < 0.0001 vs. baseline and 0-30 min hypoxia), 0.70 ± 0.30 during 0-30 min reoxygenation (*p* < 0.0001 vs. 30-180 min hypoxia), 0.89 ± 0.32 during 30-180 min reoxygenation (*p* < 0.0001 vs. 0-30 min reoxygenation), and 1.04 ± 0.27 during 3-24 h reoxygenation (*p* < 0.0001 vs. 30-180 min reoxygenation).

There were no statistically significant differences in the cFPD of the hiPSC-CMs during hypoxia or reoxygenation (Figure 4(b)). The mean cFPD was slightly longer during hypoxia but returned to baseline level during reoxygenation. On the other hand, statistically significant increase in *t*_dep_ was observed during hypoxia, and the changes were reverted during reoxygenation (Figures 4(c)–4(e)). The mean *t*_dep_ was 3.11 ± 2.62 msec during baseline, 3.12 ± 2.76 msec during 0-30 min hypoxia, 5.87 ± 4.37 msec during 30-180 min hypoxia (*p* < 0.0001 vs. baseline and 0-30 min hypoxia), 5.63 ± 5.51 msec during 0-30 min reoxygenation (*p* < 0.0001 vs. baseline and *p* < 0.01 vs. 30-180 min hypoxia), 3.73 ± 3.00 msec during 30-180 min reoxygenation (*p* < 0.0001 vs. 30-180 min hypoxia and 0-30 min reoxygenation), and 3.83 ± 2.51 msec during 3-24 h reoxygenation.

Depolarization amplitude was observed to decrease statistically significantly during hypoxia and increase during reoxygenation (Figures 4(d) and 4(e)). The mean *A*_dep_ was −0.26 ± 0.23 mV during baseline, −0.25 ± 0.21 mV during 0-30 min hypoxia, −0.13 ± 0.09 mV during 30-180 min hypoxia (*p* < 0.0001 vs. baseline and 0-30 min hypoxia), −0.12 ± 0.07 mV during 0-30 min reoxygenation, −0.17 ± 0.11 mV during 30-180 min reoxygenation (*p* < 0.0001 vs. 0-30 min reoxygenation), and −0.21 ± 0.14 mV during 3-24 h reoxygenation (*p* < 0.05 vs. 30-180 min reoxygenation).

FPP throughout the hiPSC-CM sheets was observed to slow down during hypoxia and to recover slightly during initial reoxygenation; however, at the end of the reoxygenation, the propagation was still slower compared to baseline (Figures 5(a) and 5(b)). The mean FPP time between adjacent electrodes was 3.41 ± 1.05 msec during baseline (*n*_FP_ = 1609), 3.35 ± 1.03 msec during 0-30 min hypoxia (*n*_FP_ = 603), 5.17 ± 2.74 msec during 30-180 min hypoxia (*n*_FP_ = 553, *p* < 0.0001 vs. baseline and 0-30 min hypoxia), 5.13 ± 1.94 msec during 0-30 min reoxygenation (*n*_FP_ = 487, *p* < 0.0001 vs. baseline and *p* < 0.001 vs. 30-180 min hypoxia), 4.73 ± 1.43 msec during 30-180 min reoxygenation (*n*_FP_ = 830, *p* < 0.05 vs. 30-180 min hypoxia), and 5.87 ± 2.23 msec during 3-24 h reoxygenation (*n*_FP_ = 283).

### 3.3. Hypoxia and Reoxygenation Affect hiPSC-CM Morphology and Protein Expression

hiPSC-CM morphology and protein expression were evaluated from fluorescent images of immunocytochemical staining of control, hypoxia, and hypoxia-reoxygenation samples against cardiac troponin T (cTnT), cardiac myosin binding protein C (cMyBP-C), and hypoxia inducible factor 1-alpha (HIF1-*α*). Furthermore, the protein expression of HIF1-*α* was quantified with western blot.

**Figure 6:**
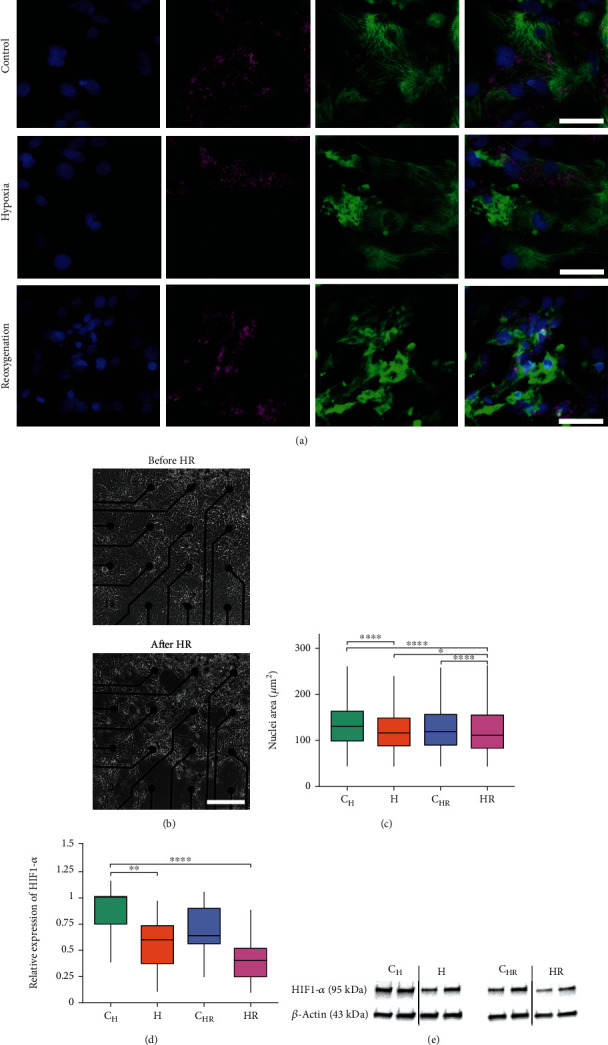
The effect of hypoxia and reoxygenation on hiPSC-CM morphology and HIF1-*α* expression. (a) Differences in the hiPSC-CM sarcomere structure (cMyBP-C, green) were observed after hypoxia and reoxygenation (scale bar 50 *μ*m), and the nucleus area was observed to decrease (counterstained with DAPI, blue). The changes in the HIF1-*α* (magenta) expression were not obvious via ICC. Confocal images of the cTnT staining of the control, hypoxia, and hypoxia-reoxygenation samples are presented in Supplementary Figure S3. (b) Phase-contrast image of the hiPSC-CMs seeded on MEA before and after hypoxia-reoxygenation treatment showed changes in the CM morphology after hypoxia-reoxygenation compared to before (scale bar 500 *μ*m). (c) Area of the hiPSC-CM nuclei was observed to decrease after hypoxia (H) and reoxygenation (HR) compared to controls (C_H_ and C_HR_) (d) Expression of HIF1-*α* from western blot was observed to decrease after hypoxia and hypoxia-reoxygenation compared to control. The blot presents two biological replicates for each condition. (e) Example blots of *β*-actin and HIF1-*α* from western blot. Full blots are presented in Supplementary Figure S4. ^∗∗^*p* < 0.01 and ^∗∗∗∗^*p* < 0.0001.

The hiPSC-CM morphology changed during hypoxia and reoxygenation, which was observed from both the fluorescent images and the phase-contrast images taken from the MEA samples before and after the hypoxia and reoxygenation (Figures 6(a) and 6(b)). The morphological changes observed from the fluorescent images included disruption of the distinct sarcomere structure of the hiPSC-CMs, as well as decrease in the nucleus area after hypoxia and reoxygenation compared to control. The mean nuclei area was 131 ± 66 *μ*m^2^ for C_H_, 119 ± 63 *μ*m^2^ for H (*p* < 0.0001 vs. C_H_), 123 ± 67 *μ*m^2^ for C_HR_, and 119 ± 70 *μ*m^2^ for HR (*p* < 0.0001 vs. C_HR_ and *p* < 0.01 vs. H). Furthermore, increased aggregation of the hiPSC-CMs after hypoxia and reoxygenation was observed from both fluorescent and phase-contrast images.

Changes in the HIF1-*α* expression were not obvious from the fluorescent images, but western blot of HIF1-*α* showed decreased expression of the protein after hypoxia and reoxygenation compared to control (Figures 6(c) and 6(d)). The mean relative expressions for HIF1-*α* were 0.89 ± 0.21 for C_H_ (*n* = 16), 0.55 ± 0.25 for H (*n* = 16, *p* < 0.01 vs. C_H_), 0.78 ± 0.58 for C_HR_ (*n* = 14), and 0.43 ± 0.26 for HR (*n* = 14, *p* < 0.0001 vs. C_H_).

### 3.4. hiPSC-CM Gene Expression after Hypoxia and Reoxygenation

**Figure 7:**
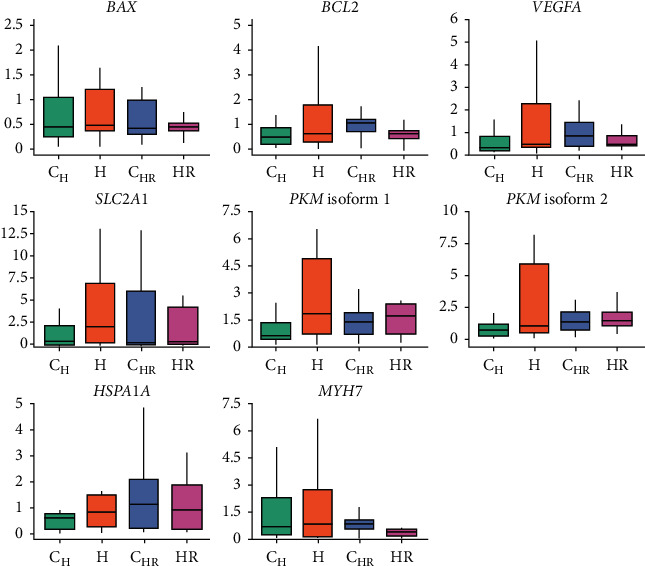
Gene expression of apoptosis-related BAX and BCL2, cellular stress-related HSPA1A and MYH7, angiogenic VEGFA and glycolysis-related SLC2A1, and PKM isoforms 1 and 2 in hiPSC-CMs after hypoxia (H: hypoxia; C_H_: control for H) and reoxygenation (HR: hypoxia; C_HR_: control for HR). There were significant intrasample and intercell line variation in the expression of the genes, and the differences in the expressions of the genes were subtle and not statistically significant.

RT-qPCR was used to evaluate the gene expression of apoptosis-related *BAX* and *BCL2*, stress markers *HSPA1A* and *MYH7*, angiogenic *VEGFA*, as well as glycolytic *SLC2A1*, and *PKM* isoforms 1 and 2 in hiPSC-CMs after hypoxia (H: *n* = 16; C_H_: *n* = 16) and reoxygenation (HR: *n* = 14; C_HR_: *n* = 16) (Figure 7). The differences in the mean relative expression of the inspected genes were subtle between the samples and were not statistically significant. Furthermore, intrasample and intercell line variation in the gene expression were large. The means and standard deviations for each gene and sample by cell line are presented in Supplementary Table S2.

The mean relative expressions of both proapoptotic *BAX* and antiapoptotic *BCL2* were slightly increased in H samples compared to C_H_ but slightly decreased in HR compared to C_HR_ samples. The mean relative expression of the *HSPA1A* and *MYH7* associated with cardiac stress was observed to increase in H samples compared to C_H_, and the *HSPA1A* expression was still elevated in HR compared to C_HR_ samples while the *MYH7* expression was decreased in HR compared to C_HR_. The mean relative expression of *VEGFA*, which is a known hypoxia response gene and related to angiogenesis, was increased in H compared to C_H_ samples. On the other hand, the expression was slightly decreased in HR compared to C_HR_ samples. The mean expressions of *SLC2A1* and *PKM* isoforms 1 and 2 related to anaerobic glycolytic metabolism were increased in H compared to C_H_ samples and were still elevated in HR compared to C_HR_, although the fold change was lower. Although there were some slight differences in the mean expressions of the sample groups, they were not statistically significant.

## 4. Discussion

In the heart, an acute ischemic event is typically due to a rupture of a plaque in a coronary artery, which can prevent blood supply to the distal cardiac tissue [32]. In addition to the sudden decrease of the oxygen level and energy sources in the cardiac tissue, the reperfusion is an acute event as well, when the blood flow is quickly restored to the heart, e.g., by angioplasty [33]. In the present study, we describe a novel model of acute cardiac ischemia and reperfusion utilizing hiPSC-CMs. We further developed our previously described platform for modeling cardiac ischemia-reperfusion [12, 20] with modifications to the 1-well assembly. Previously, we used a solid glass-made lid, with which the hypoxia and reoxygenation were reached within four hours. In the present study, we used a hollow acute lid covered with a gas permeable polyethylene membrane that allowed reaching hypoxia and reoxygenation within minutes, thus allowing the modeling of acute ischemia and reperfusion utilizing human iPSC-CMs. The hiPSC-CMs responded to hypoxia and reoxygenation with changes in their functionality, morphology, and protein expression. The observed changes were due to the pO_2_, as all samples were cultured in glucose- and serum-free medium.

Our results showed that the hiPSC-CMs responded to acute hypoxia and reoxygenation with significant changes in their electrophysiological properties (field potential, FP) measured with MEA technology. During hypoxia, the hiPSC-CM-beating frequency as well as FP amplitude decreased significantly. Furthermore, FP depolarization time increased significantly compared to baseline despite the decrease in the FP amplitude. Reoxygenation mostly reverted the changes. The changes observed in the hiPSC-CM electrophysiological properties were similar to those known to occur in adult cardiomyocytes during ischemia and reperfusion in heart. These changes occur as hypoxia decreases the ATP production of the cells, which leads to changes in the conductivity of different ions through the cell membrane and CM electrophysiological properties [34].

cFPD did not change significantly during hypoxia or reoxygenation; however, raw FPD decreased also statistically significantly during hypoxia compared to baseline (Supplementary Figure S5). In our previous study, we evaluated raw FPD values and observed similar decrease as in the present study [12, 20]. Although beating frequency is known to affect FPD [28, 35], it is also known that with very low beating frequencies; as were observed in this study, the FPD correction formulas may not work as well for the calculation of cFPD values as the formulas, such as Bazett's and Fridericia's formulas, are developed for more physiological range of beating frequencies [36]. Decrease in the action potential duration is a known effect of cardiac ischemia [34], but currently, it has not been excessively evaluated from hiPSC-CM models of cardiac ischemia-reperfusion. Thus, the present and our previous study evaluating hiPSC-CM FPD during hypoxia and reoxygenation give valuable information of the effects of these conditions on hiPSC-CM FPD.

FPP time was observed to decrease significantly during hypoxia, whereas reoxygenation improved the propagation although it did not recover to the baseline level. Decrease in electrical conduction and formation of conduction blocks in cardiac tissue is a well-known effect of ischemia and responsible for its part in generating arrhythmias, as orderly propagation of the depolarization wavefront is important in healthy cardiac contraction [37–39]. Slowing of cardiac conduction velocity is thought to be due to, e.g., changes in cell-to-cell coupling via connexin 43, but not all mechanisms are fully understood [38]. Thus, the model presented here could be used to further investigate the reasons behind this phenomenon. Furthermore, there have been studies evaluating drugs for improving conduction in cardiac tissue during and after ischemic event [40], for which the presented model could also be applied.

Changes in the hiPSC-CM morphology were similar as observed in our previous study [12, 20]. Disruption of the organized sarcomere structure was observed especially during reoxygenation, which has also been observed in other studies using hiPSC-CMs in cardiac ischemia modeling [9, 41]. Moreover, nucleus size of the hiPSC-CMs was observed to decrease after hypoxia and reoxygenation, as well as the HIF1-*α* expression, similar to our previous chronic ischemia model [12, 20]. The decrease in the nucleus size and disruption of the cell structure during hypoxia and reoxygenation could be due to cell death and consequent detachment from the cell culture substrate. As there were no significant changes in the expression of apoptotic genes after hypoxia and reoxygenation, the hiPSC-CMs likely die via necrosis, that is, a known mechanism of cell death during ischemia and reperfusion [42]. One explanation for the decrease in the HIF1-*α* expression is regulation of its expression via oxygen-independent pathways [43]. Metabolically immature hiPSC-CMs are known to have higher baseline HIF1-*α* expression in normoxic conditions [44, 45], and mechanical stress is known to induce HIF1-*α* expression as well [46]. In normoxic conditions, the hiPSC-CMs had higher spontaneous beating rate compared to hypoxia, which results in higher mechanical stress as well. The decrease in the hiPSC-CM beating rate and the mechanical stress experienced by the hiPSC-CMs during hypoxia could explain the decreased HIF1-*α* expression after hypoxia and reoxygenation.

Changes in the gene expression were very subtle and not statistically significant. Hypoxia slightly increased the expression of pro- and antiapoptotic *BAX* and *BCL2*, as well as the expression of stress markers *HSPA1A* and *MYH7*. Furthermore, a slight increase in the expressions of glycolysis-related *SLC2A1* and *PKM* isoforms 1 and 2 was observed, although the increases were not statistically significant. CMs are known to switch to glycolytic metabolism during ischemia, as the lack of oxygen in the cellular environment prevents fatty acid oxidation, the primary pathway of energy metabolism in adult CMs [47]. However, the immature hiPSC-CMs are known to rely more on aerobic glycolysis [6]. In our previous (chronic) ischemia model, significant increase in the *SLC2A1* expression was observed after hypoxia [12, 20], which could indicate that the hiPSC-CMs tried to enhance the anaerobic glycolytic metabolism by enhancing glucose uptake; however, no similar increase was observed in the present study, possibly due to significantly shorter duration of the hypoxia. However, the duration of the hypoxia was kept short, as the focus in the present study was on the acute effect of hypoxia on the hiPSC-CM functionality.

Similar systems with fast oxygen dynamics have been previously presented by a few groups [48–51]. Martewicz et al. developed a microfluidic system for modeling acute cardiac ischemia-reperfusion with relatively similar oxygen dynamics to the acute lid presented in this study. In their system, hypoxia was achieved within a few minutes and reversible alterations in the calcium dynamics of the CMs were observed during hypoxia. However, the duration of the hypoxia was short, only 5 minutes, and they used primary rat neonatal CMs instead of human CMs [48]. Another microfluidic system developed by Khanal et al. achieved 1% O_2_ within 30 minutes, which also resembles the pO_2_ dynamics of the acute lid. Moreover, the length of the hypoxia was four hours, similar to our study. But likewise to Martewicz et al., they did not use human CMs but primary pig CMs [49]. Liu et al. presented a heart-on-a-chip model with integrated extra- and intracellular bioelectronics for monitoring CM electrophysiology during acute hypoxia [50]. They achieved hypoxia within seconds, as they flushed the cell culture channel of the microfluidic chip with deoxygenated medium, and they maintained the hypoxia for 5.5 or 20 hours. However, immortalized mouse atrial CM (HL-1) cell line instead of human CMs was used in their experiments [50]. Fernández-Morales et al. on the other hand used hiPSC-CMs for evaluating calcium signaling during acute hypoxia; however, the duration of the hypoxia was only 100 seconds altogether [51]. Thus, longer term changes in the hiPSC-CMs were not evaluated in that study.

Limitations of the present study include some variability in the pO_2_ dynamics. The measurements from samples without cells showed much faster fall time of pO_2_ (6.28 ± 2.28 min) compared to the samples with cells (18.97 ± 15.86 min) (Supplementary Figure S6). Furthermore, there was more variability in the pO_2_ dynamics among the samples with cells; however, the electrophysiological responses of the hiPSC-CMs were very similar between different samples. There are several reasons for the variability between the samples, including the effect of small air bubbles and slight variations in the assembly. Air bubbles are shown to affect the local oxygen dynamics near the bubble. Therefore, the bubbles trapped on the oxygen sensor prolong the dynamics of the oxygen reading but might not affect the cells as they are not located on the sensor spot. Despite the variability in the pO_2_ dynamics, hypoxia and reoxygenation were achieved with significant speed.

hiPSC-CM immaturity is often considered as a limitation when it comes to ischemia modeling, as hiPSC-CMs resemble more fetal than adult CMs and are known to be more resistant to ischemia compared to adult CMs [6]. Furthermore, alterations in pH [51] and potassium levels [52] are known to accompany ischemia-reperfusion in cardiac tissue, but acidosis or hyperkalemia was not induced in the present study during hypoxia. However, despite these limitations, the hiPSC-CMs responded to hypoxia and reoxygenation with many electrophysiological changes that are known to occur in adult CMs during ischemia, such as decrease in beating frequency, conduction velocity, and action potential amplitude, as well as increase in depolarization time [34], and the hiPSC-CMs responded to the hypoxia and reoxygenation significantly faster compared to our previously described system [12]. In addition to the electrophysiological changes, hypoxia-reoxygenation further induced disruption of the hiPSC-CM morphology and sarcomere structure, as well as decrease in the nucleus size, which has been observed in other ischemia-reperfusion models as well [9, 41, 53].

## 5. Conclusions

In conclusion, a hiPSC-CM-based model for acute ischemia and reperfusion is presented. Hypoxia and reoxygenation are achieved significantly faster compared to our previous study [12, 20] and thus simulate rather acute than chronic ischemia. The hiPSC-CMs reproduced many known responses to hypoxia and reoxygenation, especially regarding the electrophysiological properties measured via MEA. The electrophysiological properties were comprehensively evaluated in the present study. This strongly contributes to the field of utilizing hiPSC-CMs in ischemia modeling, as the thorough evaluation of hiPSC-CM functionality during hypoxia and reoxygenation especially regarding the electrophysiology has been lacking. The presented system for modeling acute cardiac ischemia and reperfusion can be a valuable tool for drug screening and for finding new therapeutics for ischemia and reperfusion injury, as MEA provides an advantageous possibility for long-term, noninvasive, and high-throughput monitoring of the hiPSC-CM functionality [54]. Furthermore, the platform allows the study and evaluation of the mechanisms of ischemia utilizing human CMs.

## Figures and Tables

**Table 1:** TaqMan 20× assays (Applied Biosystems) used in preamplification and qPCR.

Gene	Description	Function	Assay ID
*BAX*	BCL2-associated X	Proapoptotic	Hs00180269_m1
*BCL2*	BCL2, apoptosis regulator	Antiapoptotic	Hs00608023_m1
*HSPA1A*	Heat shock protein family A member 1A	Cellular stress response	Hs00359163_s1
*MYH7*	Myosin heavy chain 7	Sarcomeric protein	Hs01110632_m1
*PKM* isoform 1	Pyruvate kinase M1	Glycolysis	Custom
*PKM* isoform 2	Pyruvate kinase M2	Glycolysis	Custom
*SLC2A1*	Solute carrier family 2, member 1/GLUT-1	Glycolysis	Hs00892681_m1
*VEGFA*	Vascular endothelial growth factor A	Angiogenesis	Hs00900055_m1
*GAPDH*	Glyceraldehyde-3-phosphase dehydrogenase	Housekeeping	Hs02758991_g1
*EEF1A1*	Eukaryotic translation elongation factor 1 alpha 1	Housekeeping	Hs00265885_g1
*TBP*	TATA-box-binding protein	Housekeeping	Hs00427620_m1

## Data Availability

All data are available on request from the authors.

## Supplementary Materials

Supplementary Materials Supplementary 1 Figure S1: characterization of UTA.11311.EURCCS hiPSC-line. Supplementary 2 Figure S2: absolute-beating frequency of hiPSC-CMs during baseline, hypoxia, and reoxygenation as beats per minute (BPM). Supplementary 3 Figure S3: images of cardiac troponin T (cTnT, green) and hypoxia inducible factor 1-alpha (HIF1-*α*, red) taken from the immunocytochemical staining of control, hypoxia, and hypoxia-reoxygenation samples. Supplementary 4 Figure S4: full blots of *β*-actin and HIF1*-α* from western blot analysis of hiPSC-CM protein expression during hypoxia and reoxygenation. Supplementary 5 Figure S5: corrected field potential duration and raw field potential duration of the hiPSC-CMs during baseline and different hypoxia and reoxygenation time points. Supplementary 6 Figure S6: oxygen partial pressure measurements from experiments without hiPSC-CMs and with hiPSC-CMs plotted in the same graph. Supplementary 7 Table S1: number of differentiation batches and samples for each cell line and experiment type. Supplementary 8 Table S2: mean relative expression of the inspected genes after hypoxia and hypoxia-reoxygenation by cell line.
