# Supplementary material for: Electrophysiological Changes of Human-Induced Pluripotent Stem Cell-Derived Cardiomyocytes during Acute Hypoxia and Reoxygenation
Source: Stem Cells Int. 2022 Dec 19;2022:9438281. doi: 10.1155/2022/9438281 (PMC9792238; doi:10.1155/2022/9438281)
Supplement: Supplementary Materials — Supplementary 1 Figure S1: characterization of UTA.11311.EURCCS hiPSC-line. Supplementary 2 Figure S2: absolute-beating frequency of hiPSC-CMs during baseline, hypoxia, and reoxygenation as beats per minute (BPM). Supplementary 3 Figure S3: images of cardiac troponin T (cTnT, green) and hypoxia inducible factor 1-alpha (HIF1-α, red) taken from the immunocytochemical staining of control, hypoxia, and hypoxia-reoxygenation samples. Supplementary 4 Figure S4: full blots of β-actin and HIF1-α from western blot analysis of hiPSC-CM protein expression during hypoxia and reoxygenation. Supplementary 5 Figure S5: corrected field potential duration and raw field potential duration of the hiPSC-CMs during baseline and different hypoxia and reoxygenation time points. Supplementary 6 Figure S6: oxygen partial pressure measurements from experiments without hiPSC-CMs and with hiPSC-CMs plotted in the same graph. Supplementary 7 Table S1: number of differentiation batches and samples for each cell line and experiment type. Supplementary 8 Table S2: mean relative expression of the inspected genes after hypoxia and hypoxia-reoxygenation by cell line. [file 9438281.f1.pdf]

Supplementary Materials

Electrophysiological changes of human induced pluripotent stem cell-derived cardiomyocytes during acute hypoxia and reoxygenation

Martta Häkli [1,*], Joose Kreutzer [2,3], Antti-Juhana Mäki [2], Hannu Välimäki [2], Reeja Maria Cherian [1], Pasi Kallio [2], Katriina Aalto-Setälä [1,4] and Mari Pekkanen-Mattila [1]

[1] Heart Group, Faculty of Medicine and Health Technology, Tampere University, Tampere, Finland
[2] Micro- and Nanosystems Research Group, Faculty of Medicine and Health Technology, Tampere University, Tampere, Finland
[3] BioGenium Microsystems Oy, Tampere, Finland
[4] Heart Hospital, Tampere University Hospital, Tampere, Finland
***** Correspondence: martta.hakli@tuni.fi

Characterization of human induced pluripotent stem cell line UTA.11311.EURCCS

Materials and Methods

UTA.11311.ERUCCS hiPSC-line was characterized as described previously [14]. Shortly, the absence of imported exogenes (*OCT4*, *SOX2*, *KLF4*, and *c-MYC*) and the expression of endogenous pluripotency genes (*OCT3/4*, *NANOG*, *SOX2*, *REX1*, and *c-MYC*) were evaluated with PCR. The expression of pluripotency markers OCT-3/4, Nanog, SSEA-4, SOX2, TRA 1-60 and TRA 1-81 on protein level was confirmed using immunocytochemistry. Furthermore, the hiPSC pluripotency *in vitro* was verified via the formation of embryoid bodies (EBs). Then, the expression of marker genes characteristic of the endoderm (*SOX17* and *AFP*), mesoderm (*KDR* and *ACTC1*), or ectoderm (*SOX1* and *PAX6*) were studied from the RNA extracted from EBs using *GAPDH* as the endogenous control. The primer sequences for pluripotency genes, marker genes of the three germ layers as well as the primary and secondary antibodies have been previously published [15]. Normal karyotype of the hiPSC-line was evaluated in the Finnish Microarray and Sequencing Centre by performing genome-wide screening for gross chromosomal abnormalities with KaryoLite BoBs (Perkin Elmer, Waltham, MA, USA) as previously described [16].

Results

In the UTA.11311.EURCCS hiPSC-line, the virally transferred exogenous pluripotency genes (*OCT4*, *c-MYC*, *SOX2*, and *KLF*) were silenced (Supplementary Figure S1A), whereas the pluripotency markers *OCT4*, *REX1*, *SOX2*, *NANOG*, and *c-MYC* were expressed at the gene level (Supplementary Figure S1B). The pluripotency of the hiPSC-line was verified using PCR to show that at least one marker form each of the three germ layers (endoderm, mesoderm and ectoderm) was expressed by the EBs formed from the hiPSCs *in vitro* (Supplementary Figure S1C). Immunocytochemistry confirmed the protein level expression of NANOG, OCT4, SOX2, SSEA4, TRA1-60 and TRA1-81 (Supplementary Figure S1D). Furthermore, the hiPSC-line expressed normal karyotype (Supplementary Figure S1E).

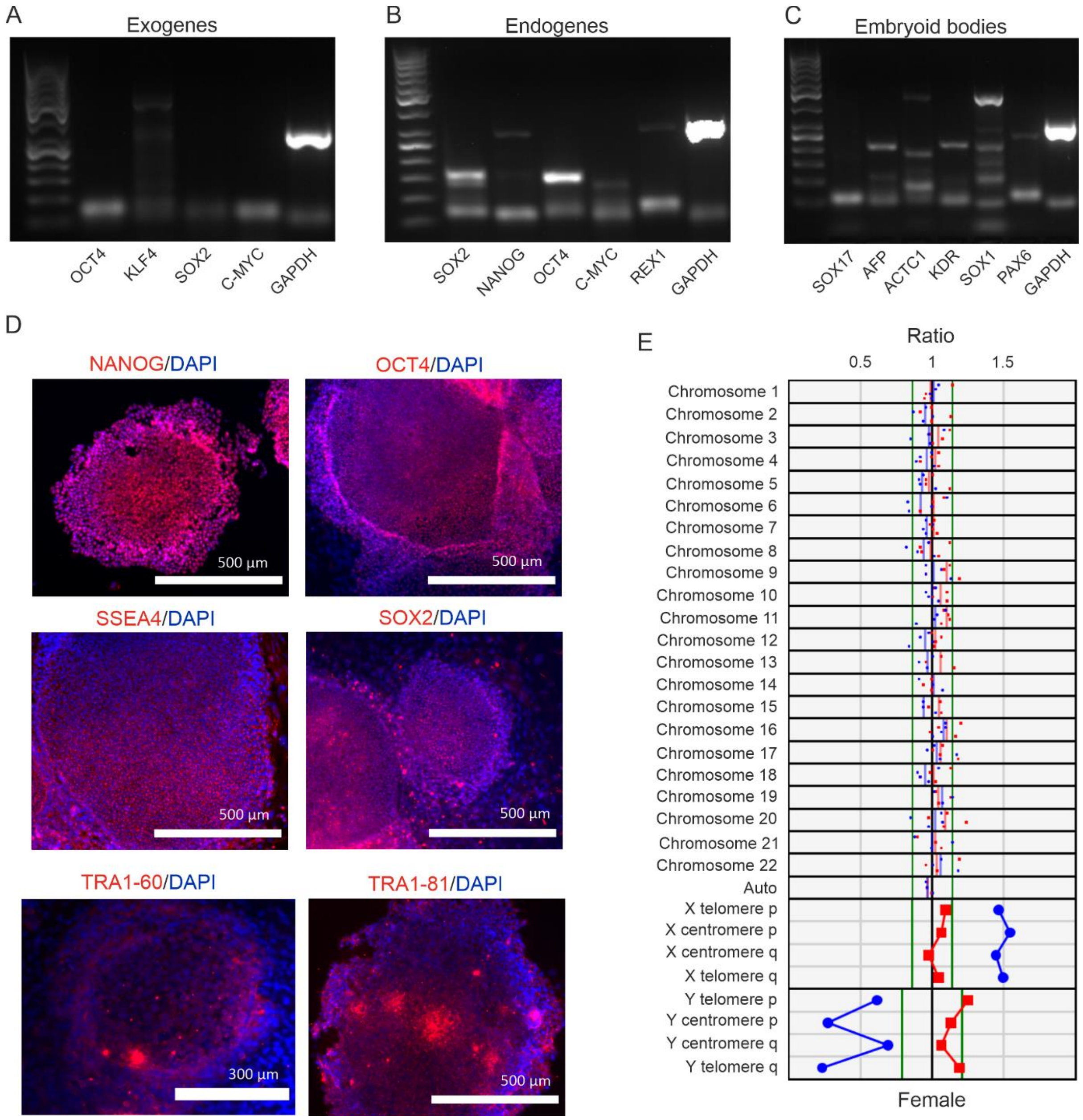

Supplementary Figure S 1. UTA.11311.EURCCS hiPSC-line pluripotency characterization by gene and protein expression, and karyotype analysis. A) The virally transferred Sendai exogenes OCT4, c-MYC, SOX2, and KLF4 were silenced in the hiPSC-line. B) The endogenous pluripotency genes OCT4, REX1, SOX2, NANOG, and c-MYC were expressed in the hiPSC-line. C) The hiPSC-line formed embryoid bodies (EBs, which) expressed at least one marker from each of the germ layers: endoderm (AFP, SOX17), mesoderm (KDR, ACTC1), and ectoderm (SOX1, PAX6). D) The protein level expression of the pluripotency markers was confirmed by immunocytochemistry. The red color indicates NANOG, OCT4, SOX2, SSEA4, TRA1-60 or TRA1-81, whereas the blue color indicates the nuclei stained using DAPI. E) Karyotype analysis of the hiPSC-line. Red and blue dots indicate chromosomal signal ratios of sample DNA against male (blue) or female (red) reference normal karyotype DNA detected by using KaryoLiteTM BoB assay. Signal from normal chromosomes should reside inside the reference area around value 1, whereas the signal residing outside the

reference are indicates abnormal karyotype. The UTA.11311.EURCCS hiPSC-line showed a normal female karyotype.

References in the main text:

[14] S. Manzini, L.E. Viiri, S. Marttila, K. Aalto-Setälä, A Comparative View on Easy to Deploy non-Integrating Methods for Patient-Specific iPSC Production, Stem Cell Rev. Reports. 11 (2015) 900–908. https://doi.org/10.1007/s12015-015-9619-3.

[15] M. Kiamehr, L.E. Viiri, T. Vihervaara, K.M. Koistinen, M. Hilvo, K. Ekroos, R. Kakela, K. Aalto-Setala, Lipidomic profiling of patient-specific iPSC-derived hepatocyte-like cells, DMM Dis. Model. Mech. 10 (2017) 1141–1153. https://doi.org/10.1242/dmm.030841.

[16] R.J. Lund, T. Nikula, N. Rahkonen, E. Närvä, D. Baker, N. Harrison, P. Andrews, T. Otonkoski, R. Lahesmaa, High-throughput karyotyping of human pluripotent stem cells, Stem Cell Res. 9 (2012) 192–195. https://doi.org/10.1016/j.scr.2012.06.008.

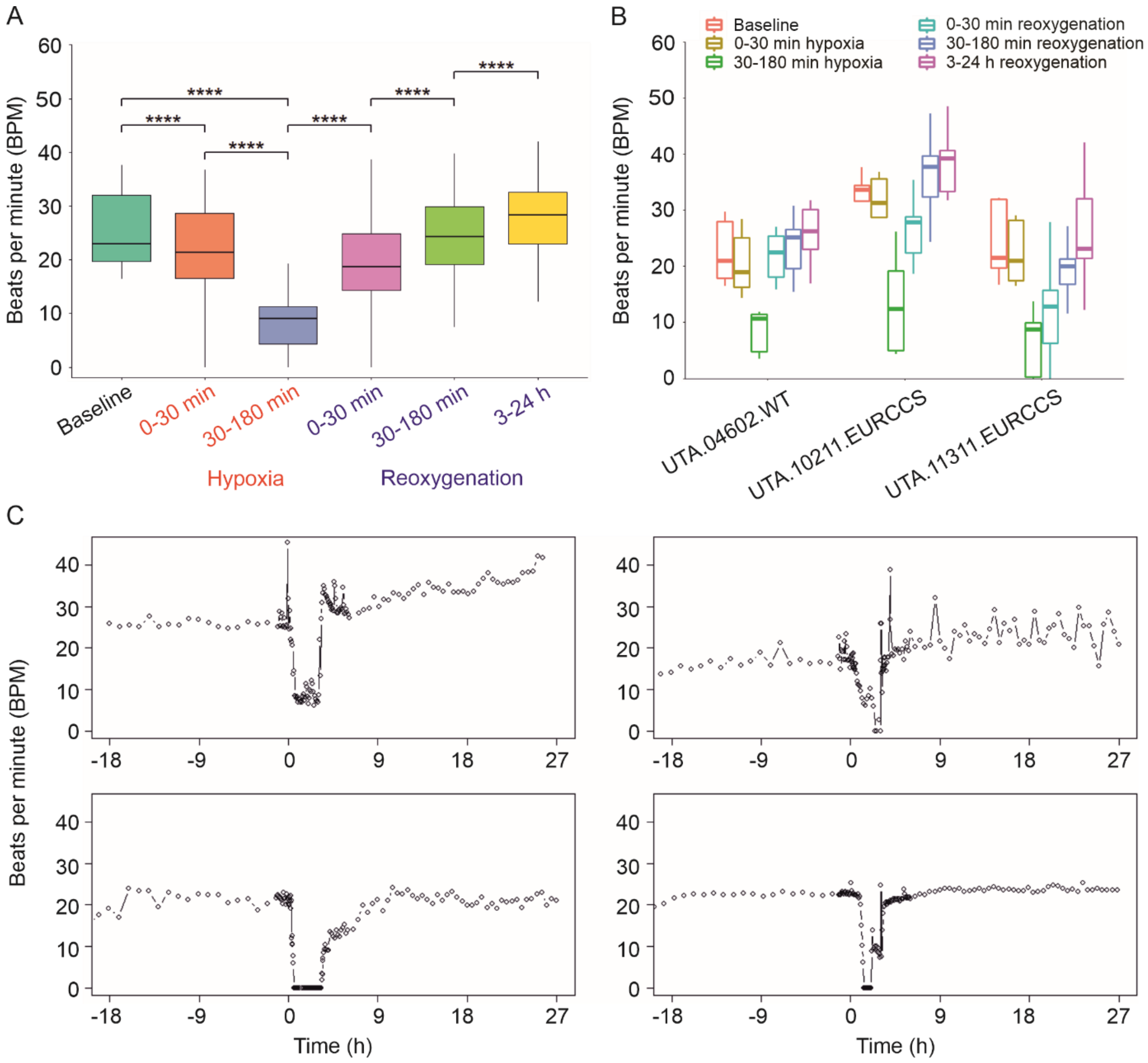

Supplementary Figure S 2. Absolute beating frequency of hiPSC-CMs during baseline, hypoxia and reoxygenation as beats per minute (BPM). A) Change in the beating frequency for all samples together. The mean beating frequency was 27.2 ± 11.9 BPM during baseline, 24.4 ± 10.3 BPM during 0-30 min hypoxia, 9.2 ± 6.8 BPM during 30-180 min hypoxia, 18.7 ± 8.9 BPM during 0-30 min reoxygenation, 24.3 ± 9.7 BPM during 30-180 min reoxygenation and 29.0 ± 8.3 BPM during 3-24 h reoxygenation. B) Change in the beating frequency by cell line. The mean beating frequency for the cell line UTA.04602.WT was 22.8 ± 5.0 BPM during baseline, 20.4 ± 4.9 BPM during 0-30 min hypoxia, 10.5 ± 6.2 BPM during 30-180 min hypoxia, 22.1 ± 3.9 BPM during 0-30 min reoxygenation, 23.3 ± 4.9 BPM during 30-180 min reoxygenation and 25.7 ± 4.8 BPM during 3-24 h reoxygenation. The mean beating frequency for the cell line UTA.10211.EURCCS was 32.0 ± 5.1 BPM during baseline, 29.7 ± 7.7 BPM during 0-30 min hypoxia, 13.5 ± 8.5 BPM during 30-180 min hypoxia, 26.6 ± 7.7 BPM during 0-30 min reoxygenation, 36.3 ± 7.8 BPM during 30-180 min reoxygenation and 38.6 ± 6.1 BPM during 3-24 h reoxygenation. The mean beating frequency for the cell line UTA.11311.EURCCS was 27.8 ± 16.2 BPM during baseline, 24.5 ± 12.9 BPM during 0-30 min hypoxia, 6.2 ± 4.7 BPM during 30-180 min hypoxia, 12.4 ± 7.5 BPM during 0-30 min reoxygenation, 19.0 ± 7.7 BPM during 30-180 min reoxygenation and 26.3 ± 7.4 BPM during 3-24 h reoxygenation. C) Line graphs of the beating frequency of the hiPSC-CMs during the 18-hour baseline measurement, 3-hour hypoxia (starting from timepoint 0) and 24-hour reoxygenation from four representative experiments. The graphs show that the hiPSC-CM functionality is very stable throughout the baseline measurement.

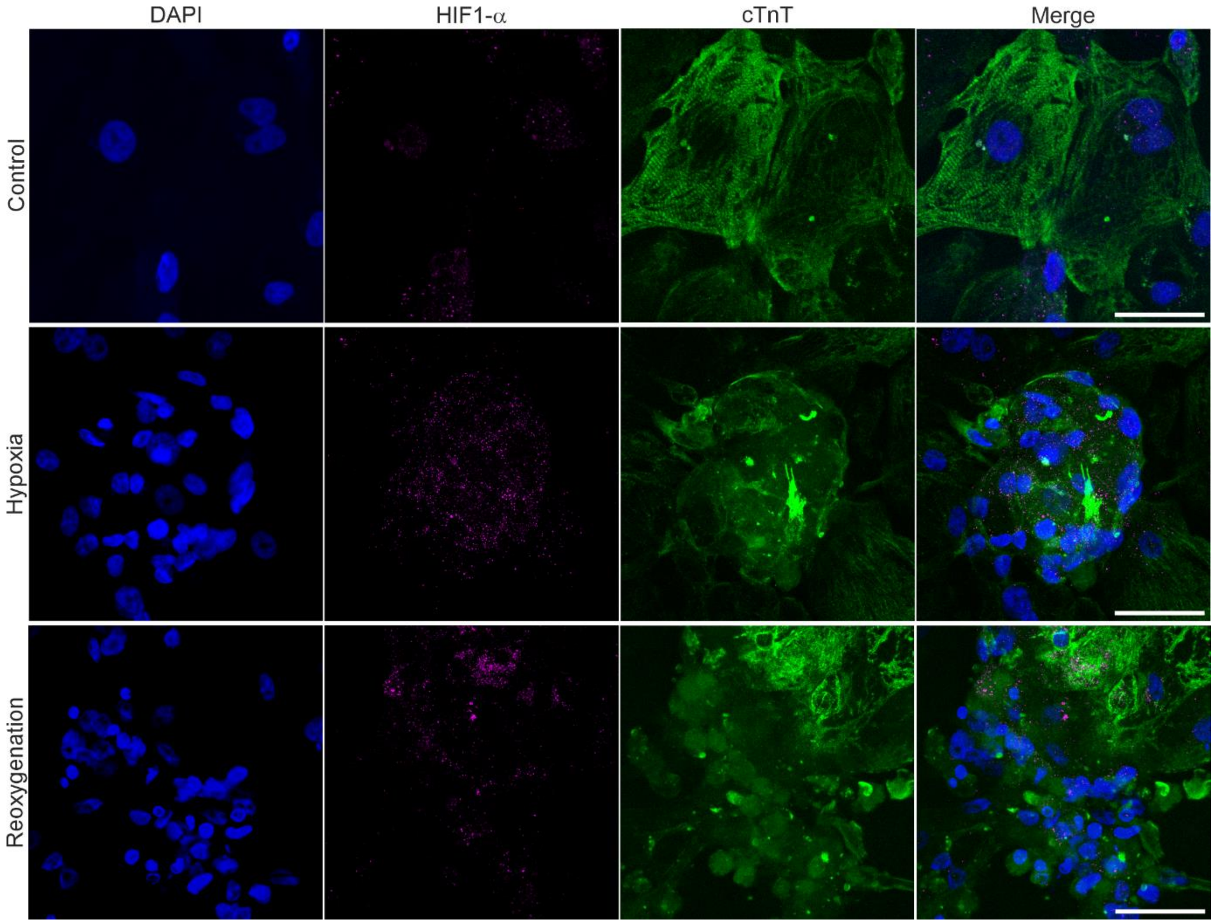

Supplementary Figure S 3. Images of cardiac troponin T (cTnT, green) and hypoxia inducible factor 1-alpha (HIF1-α, *magenta*) taken from the immunocytochemical staining of control, hypoxia and hypoxia-reoxygenation samples (scale bar 50 µm). The images show how the sarcomere structure of the hiPSC-CMs deteriorates and nuclei (DAPI, blue) size decreases during hypoxia and reoxygenation.

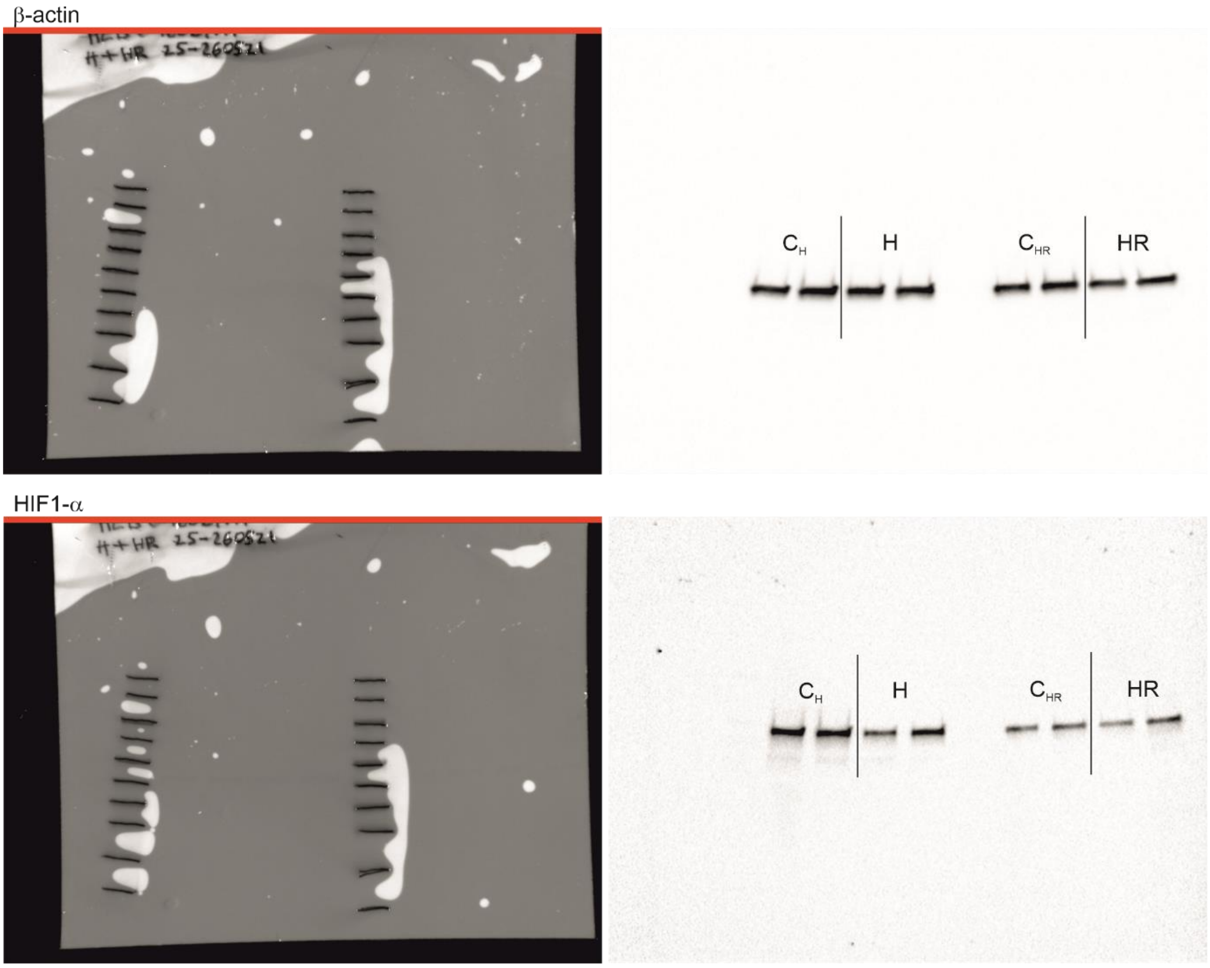

Supplementary Figure S 4. Full blots of β-actin and HIF1-α from western blot analysis of hiPSC-CM protein expression during hypoxia and reoxygenation. Samples from left to right: C_H, C_H, H, H, C_{HR}, C_{HR}, HR and HR.

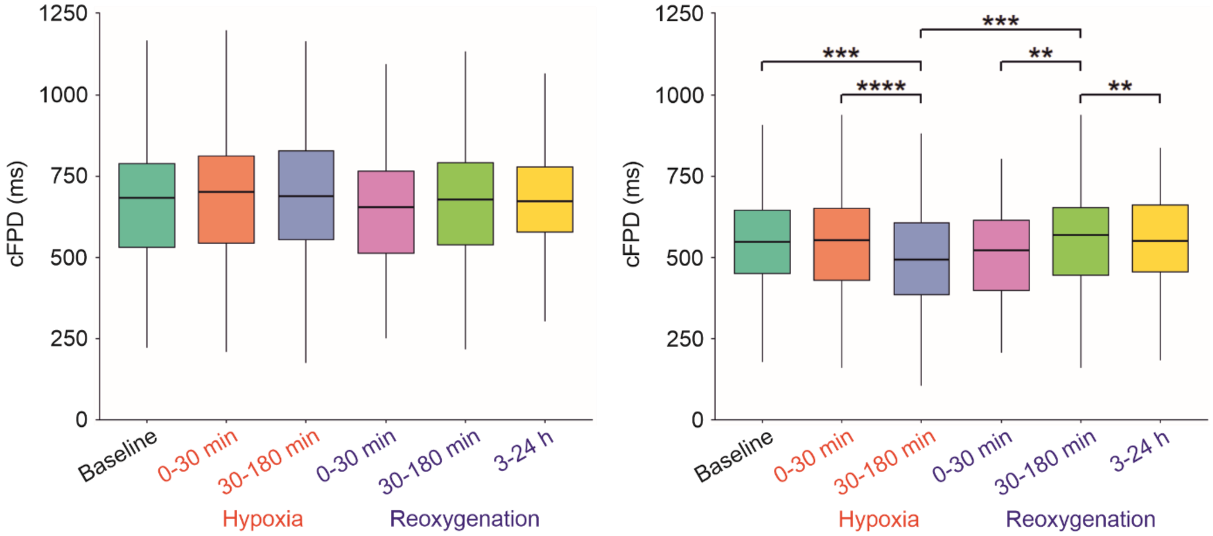

Supplementary Figure S 5. Corrected field potential duration (cFPD, calculated using Izumi-Nakaseko formula) versus raw field potential duration (FPD) values in baseline and different hypoxia and reoxygenation timepoints. ** $p < 0.01$, *** $p < 0.001$, **** $p < 0.0001$

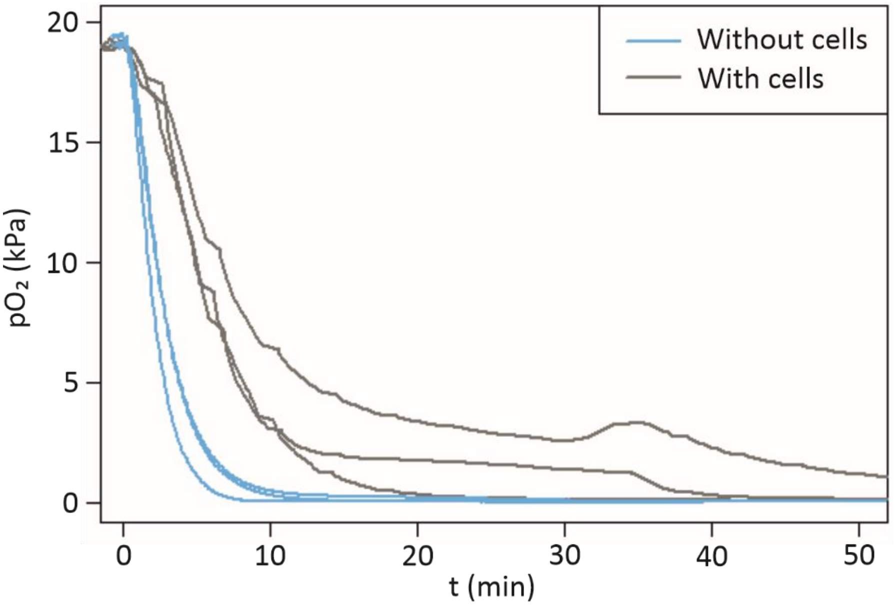

Supplementary Figure S 6. The fall time of the oxygen partial pressure measured in 1-well assembly with Acute lid was faster in the samples without cells compared to samples with cells.

Supplementary Table S 1. Number of differentiation batches and samples for each cell line and experiment type. C_H – hypoxia control; H – hypoxia; C_{HR} – hypoxia-reoxygenation control; HR – hypoxia-reoxygenation.

**Cell line**	**Differentiation batches**	**MEA**	**ICC**	**WB**	**qPCR**
UTA.04602.WT	3	7	C_H: 2<br>H: 2<br>C_{HR}: 2<br>HR: 2	C_H: 6<br>H: 6<br>C_{HR}: 6<br>HR: 6	C_H: 6<br>H: 6<br>C_{HR}: 6<br>HR: 6
UTA.11311.EURCCS	3	10	C_H: 2<br>H: 2<br>C_{HR}: 2<br>HR: 2	C_H: 6<br>H: 6<br>C_{HR}: 6<br>HR: 6	C_H: 6<br>H: 6<br>C_{HR}: 6<br>HR: 6
UTA.10211.EURCCS	3	5	C_H: 2<br>H: 2<br>C_{HR}: 2<br>HR: 2	C_H: 4<br>H: 4<br>C_{HR}: 2<br>HR: 2	C_H: 4<br>H: 4<br>C_{HR}: 4<br>HR: 2

Supplementary Table S 2. Mean and standard deviation for relative expression of the inspected genes in hiPSC-CMs after hypoxia and reoxygenation by cell line. C_H – hypoxia control; H – hypoxia; C_{HR} – hypoxia-reoxygenation control; HR – hypoxia-reoxygenation.

**Cell line**	**Sample**	***BAX***	***BCL2***	***HSPA1A***	***MYH7***	***VEGFA***	***SLC2A1***	***PKM1***	***PKM2***
UTA. 04602. WT	C_H	1.11 ± 0.56	0.67 ± 0.42	1.85 ± 2.05	2.13 ± 1.80	0.66 ± 0.69	1.90 ± 1.66	2.84 ± 3.89	1.14 ± 0.67
	H	2.00 ± 1.96	4.29 ± 7.51	4.23 ± 5.77	5.45 ± 7.27	4.58 ± 6.44	6.06 ± 4.88	13.23 ± 13.03	4.61 ± 2.80
	C_{HR}	1.11 ± 0.71	1.92 ± 1.69	2.33 ± 1.52	2.01 ± 1.06	0.72 ± 0.31	1.74 ± 3.19	3.34 ± 4.61	2.25 ± 1.86
	HR	0.57 ± 0.18	0.68 ± 0.23	1.60 ± 0.98	0.89 ± 0.65	0.50 ± 0.21	1.29 ± 2.08	3.23 ± 2.94	1.87 ± 1.41
UTA. 11311. EURCCS	C_H	0.39 ± 0.37	0.99 ± 0.69	0.12 ± 0.08	0.94 ± 0.84	0.83 ± 0.48	2.86 ± 5.27	0.84 ± 0.83	0.90 ± 0.94
	H	0.43 ± 0.41	2.11 ± 2.30	0.21 ± 0.13	1.18 ± 1.11	1.31 ± 1.06	6.29 ± 10.00	2.51 ± 2.36	3.05 ± 3.53
	C_{HR}	0.42 ± 0.43	1.12 ± 0.21	0.14 ± 0.10	0.66 ± 0.12	1.65 ± 0.58	4.24 ± 6.24	1.33 ± 0.73	2.12 ± 1.63
	HR	0.33 ± 0.23	0.70 ± 0.30	0.16 ± 0.12	0.32 ± 0.16	0.95 ± 0.44	5.39 ± 9.99	2.13 ± 2.37	2.18 ± 1.95
UTA. 10211. EURCCS	C_H	0.27 ± 0.05	0.15 ± 0.12	0.79 ± 0.20	0.08 ± 0.08	0.17 ± 0.03	0.61 ± 1.04	0.24 ± 0.32	0.25 ± 0.27
	H	0.39 ± 0.07	0.15 ± 0.15	1.38 ± 0.21	0.07 ± 0.06	0.28 ± 0.10	1.09 ± 1.89	0.43 ± 0.79	0.21 ± 0.19
	C_{HR}	0.36 ± 0.06	0.27 ± 0.16	2.99 ± 2.18	0.23 ± 0.27	0.30 ± 0.09	3.91 ± 4.81	0.58 ± 0.63	0.58 ± 0.69
	HR	0.42 ± 0.11	0.05 ± 0.01	11.77 ±2.14	0.01 ± 0.01	0.38 ± 0.04	10.18 ±14.10	0.32 ± 0.31	1.11 ± 0.02
